# Anatomical Asplenia in Cat Eye Syndrome: *An Expansion of the Disease Spectrum*

**DOI:** 10.1155/2013/218124

**Published:** 2013-04-16

**Authors:** DeepakBabu Chellapandian, Adele Schneider

**Affiliations:** ^1^Department of Pediatrics and Adolescent Medicine, Einstein Medical Center, Philadelphia, PA 19141, USA; ^2^Clinical Genetics, Einstein Medical Center, Philadelphia, PA 19141, USA

## Abstract

We report a patient with Cat eye syndrome (CES) associated with anatomical asplenia. To the best of our knowledge, there have been no prior reports of this association. Screening for asplenia in CES is potentially important, as asplenia places patients at increased risk for life-threatening bacterial infections. Hence patients with CES without a spleen may require the same routine precautions as any other asplenic patients, with penicillin prophylaxis and immunizations to protect against encapsulated organisms such as *Streptococcus pneumoniae, Haemophilus influenzae *type b, and *Neisseria meningitidis*.

## 1. Introduction

Cat eye syndrome (OMIM 115470) is a rare chromosomal disorder presenting as a clinically recognizable pattern of congenital abnormalities, first described in 1965 by Schachenmann et al. The classical features are ocular coloboma, anorectal, heart, and renal malformations, ears with pre-auricular tags and/or pits and variable intellectual disabilities [1, 2]. The name “cat eye” was introduced because the iris coloboma resembles the pupil shape of cats. CES is often associated with significant phenotypic variability, ranging from patients with almost normal phenotype to those with severe abnormalities [1]. None of the features are consistently present. Only 41% of CES patients have the combination of iris coloboma, anal anomalies, and preauricular pits/tags [1]. Thus, many patients cannot be identified as having CES by phenotype alone. Here, we describe a male infant with clinical features of cat eye syndrome, confirmed by cytogenetic and fluorescence in situ hybridization (FISH) analysis, who presented with anatomical asplenia. 

## 2. Case Report

The proband is a full term male, born to a gravida 2 para 1 mother by Caesarian section. At the patient's birth, the mother and father's ages were 22 and 20, respectively. The pregnancy was a result of natural conception and uncomplicated by any teratogens. The prenatal ultrasound was reported as normal. The parents were healthy and unrelated. The mother has a healthy 3-year-old daughter from a previous relationship. Review of the family history did not reveal any other individuals with developmental delay or congenital malformations.

**Figure 1:**
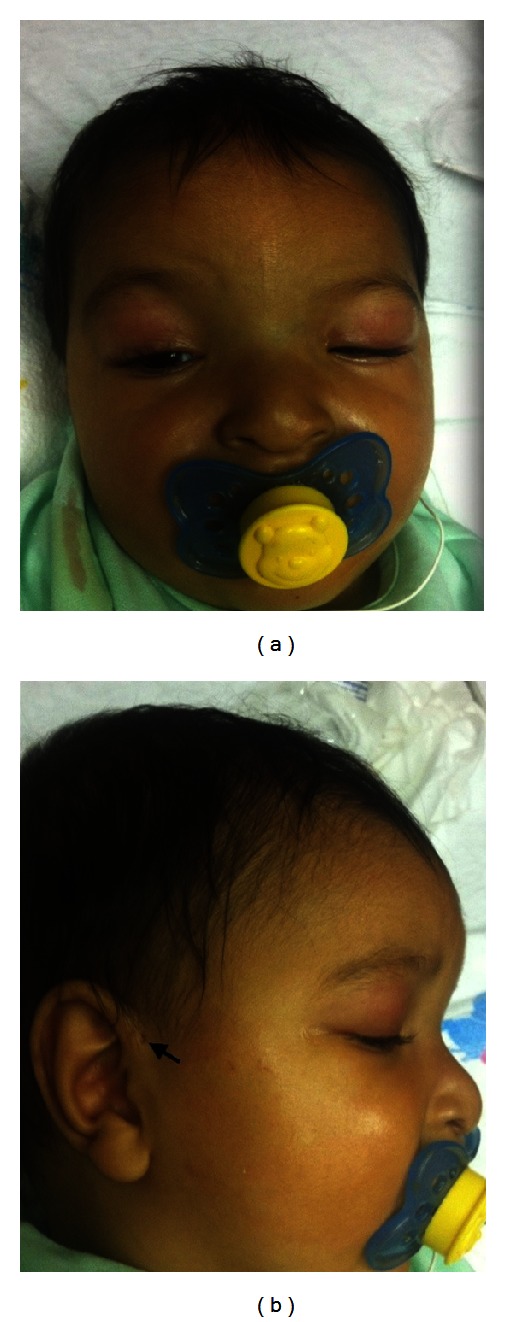
Clinical presentation of CES: (a) hypertelorism with facial asymmetry and (b) preauricular pit (arrow).

The patient was referred to our center from an outside hospital soon after birth for surgical management of imperforate anus. His birth weight was 3345 grams (75th centile), length was 52.5 cm (>90th centile), and head circumference was 35.5 cm (90th centile). The initial physical examination on admission showed the following dysmorphic features: downslanting palpebral fissures, hypertelorism (Figure 1(a)) with interpupillary distance of 5 cm and inner canthal measurement of 3 cm (both are above 97th centile for age), mild facial asymmetry (right side > left side), slight difference between the size of the ears (<0.5 cm), anteverted and low set ears with bilateral preauricular pits (Figure 1(b)), micrognathia, and bilateral 5th finger clinodactyly. His initial detailed ophthalmological examination showed iris coloboma with normal retinal vascularization. A grade II/VI systolic murmur, imperforate anus with perineal fistula, left renal pelviectasis, tethered cord, and partial fusion of S1 and S2 vertebra were other noticeable abnormalities detected at birth. Echocardiogram revealed a patent foramen ovale, small ASD and a restrictive VSD with small PDA and a normal systolic function. The patient underwent surgical correction for imperforate anus and received postoperative care in the neonatal intensive care unit. He was discharged home on amoxicillin prophylaxis for his renal abnormality and scheduled for voiding cystourethrogram (VCUG) at 1 month of age. His initial feeding intolerance resolved before discharge and he was sent home on full enteral feeds. He also had a normal hearing screen test at discharge.

Two weeks after discharge from the hospital, the infant was readmitted for respiratory syncytial virus (RSV) associated bronchiolitis for which he received supportive care. By day 3 of the hospital stay he started having multiple spikes of high-grade fever along with signs of early septic shock. His septic workup showed leukocytosis (28,700 cells/cu·mm) with bandemia and markedly elevated C-reactive protein (16 mg/dL). He was stabilized with intravenous fluids and started empirically on cefotaxime, vancomycin, and ampicillin to cover broad spectrum of gram-negative and gram-positive organisms along with Listeria monocytogenes. Persistent fever spikes prompted us to perform an abdominal ultrasound as a measure to detect the source of fever. The ultrasound showed dilated left pelvicalyceal system and absence of the spleen in the left upper quadrant. To confirm the findings of asplenia, liver-spleen scan was done using radionuclide SPECT scan, which confirmed anatomical asplenia with no evidence of any accessory spleen along with presence of a normal liver. His fever defervesced and he was clinically stable after 48 hours of starting antibiotics. The antibiotics were discontinued after 7 days following the negative blood cultures. While considering a possibility of immunodeficiency, a complete immunological workup including T-cell subset studies and a complete immunoglobulin profile was performed which were within normal limits. His newborn genetic and metabolic screening test was essentially normal. The patient was discharged home on amoxicillin prophylaxis in view of asplenia. He was followed periodically by his general pediatrician and also other specialties including genetics and immunology. 

At 7 months of followup, he was developmentally appropriate for his age with up-to-date immunization status including pneumococcal conjugate and *Hemophilus influenza* type b vaccine. His cardiac septal defects spontaneously resolved and he had normal biventricular function with mild pulmonary branch stenosis. MRI of the spine done at 9 months of age revealed a stable lipoma of the filum terminale with segmentation anomalies of L5-S1 vertebrae for which he underwent a neurosurgical procedure including sacral laminectomy. The tethering of the spinal cord that was diagnosed at birth was not appreciated during this surgical procedure. He had an uneventful postoperative period.

## 3. Cytogenetic Analysis

**Figure 2:**
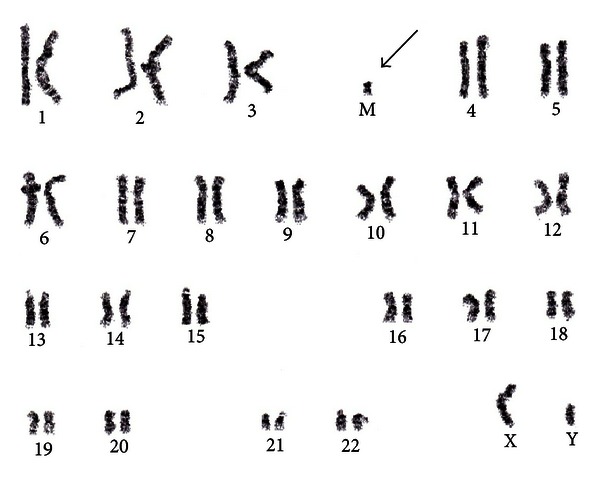
Patient's karyotyping showing 47, XY, isodicentric supernumerary marker chromosome (M) (arrow).

The patient's karyotype showed a male karyotype 47 XY, isodicentric (22)(q11.2) (Figure 2), and chromosome single nucleotide polymorphism (SNP) microarray analysis showed 2.60 MB tetrasomy of proximal 22Q11.1→22Q11.21 consistent with cat eye syndrome {arr  22q11.1q11.21(14,435,171–17, 035, 860)X  4}. The four-copy gain of this region is typically associated with a supernumerary bisatellited marker. 

## 4. Discussion

Cat eye syndrome is a rare malformation syndrome with an estimated incidence between 1 : 50,000 and 1 : 150,000 [3]. In 1878, Haab first described the association of iridochoroidal coloboma, left renal hypoplasia, and anal atresia in a three-day-old infant who died from rectal rupture. In 1965 Schachenmann and his colleagues associated coloboma of the iris and anal atresia with a small extra marker chromosome [4].

The phenotypic variability of CES makes it hard to define clinical criteria for this disorder. A preauricular tag or pit is the most consistent feature in CES [1]. Many CES patients cannot be identified as having CES by their phenotype alone. The presence of the inverted duplicated chromosome 22 has been the most valuable diagnostic criterion. Many cases showing incomplete expression have been reported. This is the first report of asplenia in CES and expands the spectrum of anomalies to include asplenia. 

We also considered other causes of asplenia to ensure that there were no findings of a second syndrome. Congenital asplenia is often associated with complex visceral defects, as part of the so-called visceroatrial heterotaxy syndromes. Perhaps the best known among these heterotaxy syndromes is the asplenia/polysplenia syndrome, which was fully documented by Ivemark in 1955 [5]. The majority of cases are sporadic, although familial cases have been described. Mutations have been identified in different genes controlling left-right laterality. They also suffer from various anomalies of the heart and great vessels. The spleen is almost always affected in patients with visceral heterotaxy that may be composed of a cluster of small splenunculi, a large spleen, and several small ones, or it may be multilobed (polysplenia) [6]. In a study by Van Praagh et al. out of the 109 cases of visceral heterotaxy with congenital heart disease seen at autopsy, 53% had asplenia, 42% had polysplenia, and 5% had a single, normal-sized spleen that was abnormally located in the upper right side of the abdomen [7]. He also described other abnormalities in the lung and liver in the asplenia group. The cardiac defect in our patient is likely a component of the CES phenotype and the absence of other organ-specific abnormalities reasonably rules out heterotaxy syndrome. 

Another congenital condition with asplenia is the isolated congenital asplenia, which is more common than was previously thought, with autosomal dominant inheritance [8]. In contrast to the heterotaxy syndrome, it lacks other developmental anomalies, particularly those of the cardiovascular system. There have been case reports of asplenia discovered after the death of an otherwise healthy adult or child from overwhelming sepsis with encapsulated organisms [9]. The combination of Howell-Jolly bodies and thrombocytosis in the blood of an individual without a palpable spleen or an abdominal scar suggests the diagnosis of congenital asplenia. 

According to Rosias et al. one of the most common causes of early death in CES patients is sepsis and bronchopneumonia [2]. Placing the asplenic CES patients on long-term penicillin or amoxicillin prophylaxis (until age 5 years) may be protective against various life-threatening infections. Vaccination should be performed in these children according to the recommended pediatric dosage and vaccine types with special consideration made for children less than 2 years of age [10]. Children less than 2 years of age should receive pneumococcal conjugate vaccine (PCV-13 if available) at the usual ages recommended for children (2, 4, 6, and 12–15 months of age). Older children and adults should receive polyvalent pneumococcal vaccine (PPV-23) with booster approximately every 5 years starting from the first dose. Quadrivalent meningococcal vaccine should be given to all asplenic individuals ≥2 years of age with boosters every 3–5 years. Hemophilus influenza vaccine should be given at the time of routine vaccination for younger children until 2 years of age. Because approximately 20% of invasive pneumococcal infections in asplenic patients are nonvaccine strains, a high degree of vigilance remains essential during the long-term followup [11].

## 5. Conclusion

Here we report a Hispanic patient, who showed tetrasomy of chromosome 22q11.2 resulting from a supernumerary isodicentric marker chromosome. Though our patient did not present with all the characteristic features of CES, the finding of asplenia in CES is an undiscovered association so far. We strongly recommend screening CES patients for asplenia in order to be more vigilant in the prevention of life-threatening infections. Examination of a blood smear for Howell-Jolly bodies is a simple procedure and may be lifesaving. Imaging to determine whether a spleen is present should follow identification of Howell-Jolly bodies on the blood smear. Given the fact that asplenia can be a potential risk factor for overwhelming sepsis, early recognition of asplenia in CES patients would be helpful in avoiding fulminant sepsis, septic shock, and death related to bacteremia with the potential for overall improvement in the survival of these patients.
